# The role of cardiovascular magnetic resonance in stratifying paravalvular leak severity after transcatheter aortic valve replacement: an observational outcome study

**DOI:** 10.1186/s12968-014-0093-x

**Published:** 2014-12-05

**Authors:** Gregory R Hartlage, Vasilis C Babaliaros, Vinod H Thourani, Salim Hayek, Christina Chrysohoou, Nima Ghasemzadeh, Arthur E Stillman, Stephen D Clements, John N Oshinski, Stamatios Lerakis

**Affiliations:** Department of Medicine, Division of Cardiology, Structural Heart and Valve Center, Emory University School of Medicine, Atlanta, Georgia; Department of Radiology and Imaging Science, Emory University School of Medicine, Atlanta, GA Georgia; Department of Surgery, Division of Cardiothoracic Surgery, Structural Heart and Valve Center, Emory University School of Medicine, Atlanta, Georgia; Department of Biomedical Engineering, Georgia Institute of Technology/Emory University, Atlanta, Georgia

**Keywords:** Cardiovascular magnetic resonance imaging, Paravalvular regurgitation, Prognosis, Transcatheter aortic valve implantation

## Abstract

**Background:**

Significant paravalvular leak (PVL) after transcatheter aortic valve replacement (TAVR) confers a worse prognosis. Symptoms related to significant PVL may be difficult to differentiate from those related to other causes of heart failure. Cardiovascular magnetic resonance (CMR) directly quantifies valvular regurgitation, but has not been extensively studied in symptomatic post-TAVR patients.

**Methods:**

CMR was compared to qualitative (QE) and semi-quantitative echocardiography (SQE) for classifying PVL and prognostic value at one year post-imaging in 23 symptomatic post-TAVR patients. The primary outcome was a composite of all-cause death, heart failure hospitalization, and intractable symptoms necessitating repeat invasive therapy; the secondary outcome was a composite of all-cause death and heart failure hospitalization. The difference in event-free survival according to greater than mild PVL versus mild or less PVL by QE, SQE, and CMR were evaluated by Kaplan-Meier survival analysis.

**Results:**

Compared to QE, CMR reclassified PVL severity in 48% of patients, with most patients (31%) reclassified to at least one grade higher. Compared to SQE, CMR reclassified PVL severity in 57% of patients, all being reclassified to at least one grade lower; SQE overestimated PVL severity (mean grade 2.5 versus 1.7, p = 0.001). The primary and secondary outcomes occurred in 48% and 35% of patients, respectively. Greater than mild PVL by CMR was associated with reduced event-free survival for the primary outcome (p < 0.0001), however greater than mild PVL by QE and SQE were not (p = 0.83 and p = 0.068). Greater than mild PVL by CMR was associated with reduced event-free survival for the secondary outcome, as well (p = 0.012).

**Conclusion:**

In symptomatic post-TAVR patients, CMR commonly reclassifies PVL grade compared with QE and SQE. CMR provides superior prognostic value compared to QE and SQE, as patients with greater than mild PVL by CMR (RF > 20%) had a higher incidence of adverse events.

## Background

Transcatheter aortic valve replacement (TAVR) has transformed the contemporary management of patients with severe aortic stenosis and high surgical risk [1,2]. Despite extensive pre-procedure evaluation, greater than mild paravalvular leak (PVL) occurs in over 10% of patients undergoing TAVR and is associated with worse short and long term outcomes [3,4]. Echocardiography, the standard non-invasive method of imaging PVL, often has limited utility due to multiple eccentric regurgitant jets [5]. Acoustic shadowing from the valve stent and native aortic valve calcification may further complicate the estimation of PVL. The Valve Academic Research Consortium II (VARC II) has proposed a semi-quantitative method of PVL classification by the extent of circumferential involvement relative to the prosthetic valve stent on two-dimensional echocardiography [6], which is yet to be validated.

Accurate identification of post-TAVR PVL by cardiac imaging is essential, as symptoms from PVL may be difficult to clinically differentiate from symptoms related to other common causes of heart failure, such as systolic and diastolic dysfunction. Cardiovascular magnetic resonance (CMR) flow assessment provides accurate and reproducible quantification of valvular regurgitation [7,8]. The direct measurement of forward and reverse flow volumes by CMR facilitates the calculation of the aortic regurgitant fraction (RF) for severity classification. Small studies have demonstrated the feasibility of CMR for evaluation of post-TAVR PVL [9-11], however, these studies did not address diagnostic and prognostic value in symptomatic patients. The purpose of our study was to evaluate CMR in post-TAVR patients in whom there was concern for clinically significant PVL. In addition to PVL classification, we assessed the ability of CMR findings to predict outcomes at one year post-imaging.

## Methods

### Study population

The study was approved and performed in accordance with the regulations of the university’s institutional review board (Emory University Hospital, Atlanta, Georgia). We retrospectively reviewed all patients at our center from 2009 to 2013 that underwent CMR post-TAVR. Patients had been implanted with either a balloon-expandable or self-expanding transcatheter aortic prosthesis. We included all patients undergoing CMR for evaluation of heart failure symptoms persisting or recurring after TAVR, which were potentially related to PVL seen on transthoracic echocardiography (TTE). A detailed medical history, laboratory values and TTE findings were collected to establish baseline demographics prior to TAVR and prior to CMR.

### Echocardiography

**Figure 1:**
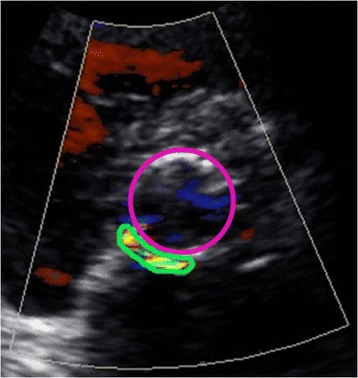
**Semi-quantitative echocardiographic (SQE) measurement of paravalvular leak (PVL) using circumferential extent of PVL in the parasternal short-axis by the Valve Academic Research Consortium II method.** The green circle represents the circumference of the paravalvular leak. The purple circle represents the circumference of the transcatheter valve stent frame. The circumferential extent is the sum of the paravalvular leak jet circumference(s) divided by the valve circumference (mild <10%, moderate 10-30%, severe >30%).

TTE was performed on a Phillips iE33 (Leiden, The Netherlands) or General Electric Vivid 7 (Milwaukee, Wisconsin) as the initial test for the evaluation of PVL. PVL was classified qualitatively by visual assessment at the point of care, as well as semi-quantitatively by VARC II criteria post-hoc. Qualitative echocardiography (QE) included visual estimation of the width and area of the color Doppler jet in the left ventricular outflow tract in the parasternal short and long-axis views and was graded mild (jet width < 25% of left ventricular outflow tract width), moderate (jet width 25 to 65% of left ventricular outflow tract width), and severe (jet width >65% of left ventricular outflow tract width). For semi-quantitative echocardiography (SQE), the VARC II PVL classification scheme was employed using the circumferential extent of the regurgitant jet(s) in the parasternal short-axis (the sum of the PVL jet circumferences divided by the valve circumference; graded as mild <10%, moderate 10 to 30%, severe >30%; see Figure 1). Suprasternal notch and subcostal views of the descending aorta and parasternal short and long-axis view of the right ventricular outflow tract and main pulmonary artery were consistently attempted.

### Cardiovascular magnetic resonance acquisition

**Figure 2:**
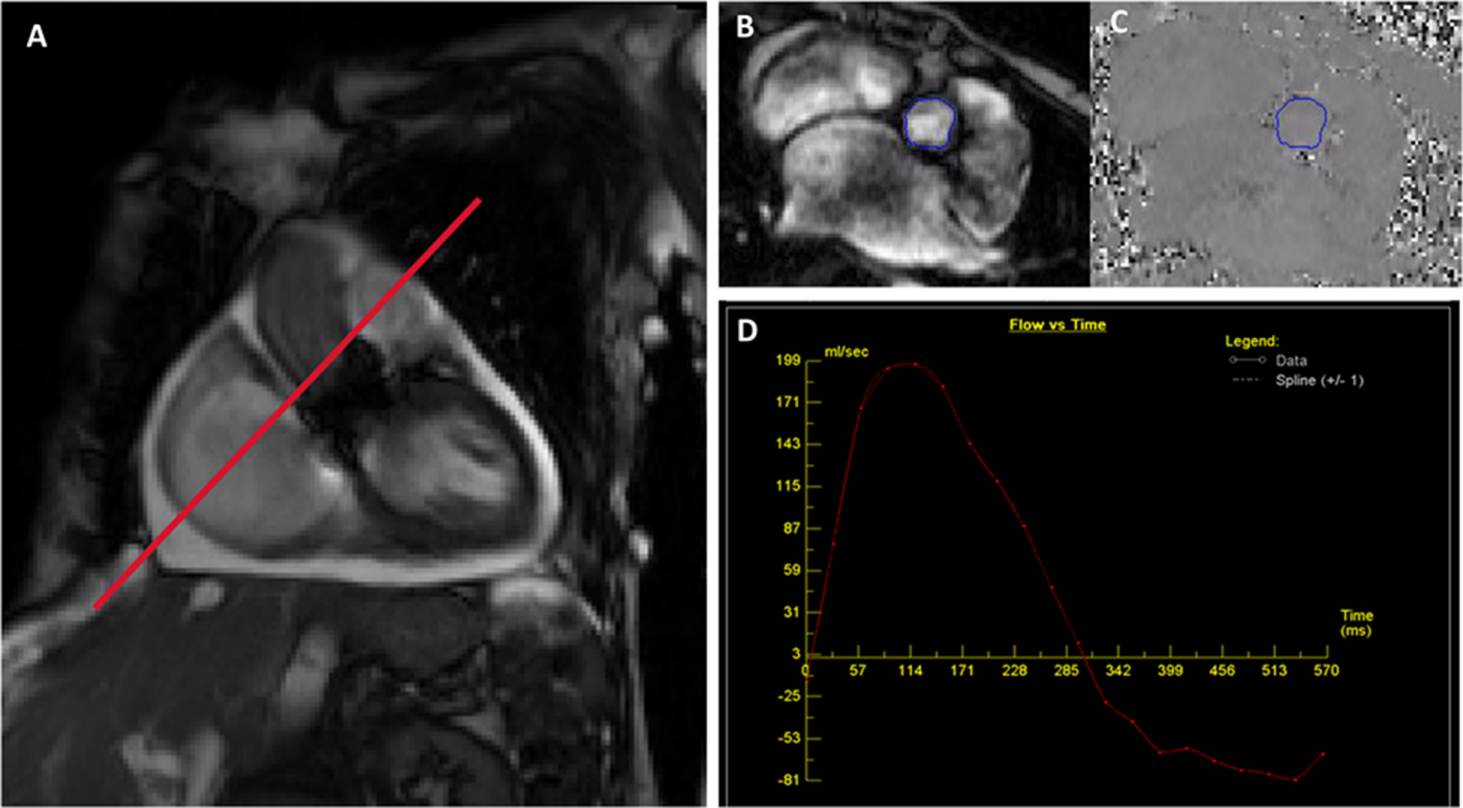
**Measurement of paravalvular leak (PVL) regurgitant fraction (RF) by cardiovascular magnetic resonance (CMR).** The positioning of the scan plane is demonstrated for the balloon-expandable prosthesis in the aortic root 2-3 mm above the valve stent frame **(A)**. The regions of interest are traced on the magnitude images (anatomical scan; **B**) and the phase images (flow scan; **C**). The regions of interest include the entire intra-luminal, cross sectional area of flow just above the transcatheter valve. The flow through the region of interest is calculated throughout the cardiac cycle **(D)**, with the area under the curve (above baseline) representing forward flow volume and the area above the curve (below baseline) representing reverse flow volume. The aortic regurgitant fraction was calculated by dividing the reverse flow volume by the forward flow volume (mild ≤ 20%, moderate 21-39%, severe ≥40%).

CMR was performed on a Siemens Avanto 1.5 T scanner (Erlangen, Germany) using a 5 element phased array coil. Velocity-encoded phase-contrast magnetic resonance (PCMR) imaging was utilized in the ascending aorta for flow quantification [12]. The scan plane was placed perpendicular to the long-axis of the proximal ascending aorta 2-3 mm above the valve stent frame for through-plane flow measurement in patients with a balloon-expandable prosthesis (see Figure 2A) and at a similar position through the non-ferromagnetic stent frame in patients with a self-expanding prosthesis. Free-breathing acquisitions were used preferentially, including patients with irregular heart rhythms, with breath-hold acquisitions reserved for patients with respiratory image artifacts due to irregular breathing. Electrocardiographic triggering was used to obtain 20 frames per cardiac cycle for patients with a regular heart rhythm, while free-breathing real time acquisitions with multiple signal averaging was used for patients with an irregular heart rhythm; the slice thickness was 6 mm. The VENC was initially set at 200–250 cm/s with routine VENC optimization.

### Cardiovascular magnetic resonance analysis

For the assessment of aortic RF, the cross sectional area of the aorta was traced throughout the cardiac cycle on each separate magnitude image to define regions of interest (see Figure 2B). The forward and reverse flow volumes were calculated within the corresponding region of interest on the phase image (see Figure 2C) by offline analysis using Siemens Argus software (Erlangen, Germany). Background phase offset correction was done by using a region of static tissue near the ascending aorta. Aortic RF was calculated as: reverse aortic flow volume divided by forward aortic flow volume; see Figure 2D. CMR PVL severity was classified by regurgitant fraction (RF; mild ≤ 20%, moderate 21 to 39%, severe ≥40%).

### PVL classification comparison

The classification of PVL was compared using three modalities in all patients: QE, SQE, and CMR (used as the gold standard for this study). By convention, PVL was classified as 1 (mild), 2 (moderate) and 3 (severe) for analysis. Inter-method agreement of classification was analyzed within each patient and for the whole group. We documented reclassification of PVL grade by CMR relative to QE and SQE. PVL severity was considered downgraded if the CMR classification was ≥1 grade lower and upgraded if ≥1 grade higher as compared to each TTE method.

### Outcomes

Study outcomes were determined by retrospective chart review. All patients in this analysis were enrolled in prospective TAVR trials or registries with long-term follow-up and data collection at our clinic. Follow-up included detailed interval histories including any hospital admissions or procedures taking place at Emory University Hospital or outside healthcare facilities. Any patient deaths not taking place at Emory University Hospital were reported to clinic study personnel and documented in the chart, as well. The primary composite outcome was the combination of all-cause death, heart failure hospitalization, and intractable heart failure symptoms necessitating repeat invasive therapy at one-year follow-up. The secondary composite outcome included the combined endpoints of all-cause death and heart failure hospitalization. Intractable heart failure included New York Heart Association (NYHA) class III and IV symptoms despite aggressive oral diuretic and/or vasodilator therapy. Repeat invasive therapy for significant PVL included repeat balloon valve dilation, transcatheter PVL plug placement, transcatheter valve-in-valve placement, or surgical valve replacement. Heart failure hospitalization included any inpatient admission for congestive symptoms including worsening dyspnea and peripheral edema during which the patient was treated with intravenous diuretic and/or vasodilator therapy.

### Statistics

Statistical analysis was performed with SPSS version 20 (Chicago, Illinois) and SAS version 9.3 (Cary, North Carolina). Descriptive results were expressed as numbers and percentages, while continuous variables were expressed as mean ± standard deviation (unless otherwise stated). Continuous variables were analyzed by the independent-samples t-test. Categorical variables were analyzed by the Fisher’s exact test. PVL classification by each method was compared within patients by Spearman rho bivariate correlation and the groups were compared by the paired-samples Wilcoxon rank-sum test. Baseline risk was compared across mild, moderate, and severe PVL by CMR with the Kruskal-Wallis test to evaluate for significant differences between the groups. The difference in event-free survival according to greater than mild PVL versus mild or less PVL by QE, SQE, and CMR were evaluated by Kaplan-Meier survival analysis with Mantel-Cox log-rank. Inter-observer variability was assessed with intraclass correlation for continuous variables and Cohen’s kappa coefficient for dichotomous classification (severity greater than mild versus mild or less). Statistical significance was determined by p < 0.05 on two-tailed analysis.

## Results

### Patients

CMR was performed in 23 post-TAVR patients with NYHA class III-IV symptoms (median 6 days [interquartile range 2–100 days] post-TAVR and median 2 days [interquartile range 0–7 days] after index echocardiogram). The baseline Society for Thoracic Surgeons Predicted Risk of Mortality score was not significantly different among patients with mild, moderate, and severe PVL by CMR (p = 0.26). Baseline patient characteristics at the time of TAVR are presented in Table 1 and interval clinical and imaging characteristics at the time of CMR in Table 2. Patients with significant PVL with RF > 20% on CMR were more likely to have atrial fibrillation at baseline. At the time of CMR, patients with a RF > 20% had higher serum creatinine, higher serum brain-natriuretic peptide levels, larger PVL circumferential extent, and higher prevalence of other valve dysfunction of at least moderate degree (mitral and tricuspid regurgitation). Otherwise, there were no significant baseline differences at the time of TAVR or at the time of CMR in those with RF > 20% versus RF ≤ 20%.

**Table 1:** Baseline patient characteristics at TAVR*

	**All (n = 23)**	**RF < 20% (n = 11)**	**RF > 20% (n = 12)**	**P value**
Male	16 (70)	6 (55)	10 (83)	0.19
Age (mean ± SD)	83 ± 6	83 ± 7	82 ± 5	0.86
NYHA class (mean ± SD)	3.2 ± 0.7	3.1 ± 0.7	3.3 ± 0.8	0.61
STS-PROM (%; mean ± SD)	6.9 ± 2.4	6.4 ± 2.0	7.4 ± 2.7	0.33
Body mass index (kg/m^2^)	25 ± 4	25 ± 4	24 ± 4	0.64
Comorbidities				
Coronary artery disease	13 (57)	7 (64)	6 (50)	0.68
CABG	7 (30)	2 (18)	5 (42)	
PCI	8 (35)	4 (36)	4 (33)	
Myocardial infarction	7 (30)	3 (27)	4 (33)	
Atrial fibrillation	10 (44)	2 (18)	8 (67)	0.04
Diabetes mellitus	10 (44)	5 (46)	5 (42)	1.00
Hypertension	21 (91)	10 (91)	11 (92)	1.00
Serum creatinine >1.5 mg/dl	8 (35)	2 (18)	6 (50)	0.19
COPD	8 (35)	3 (27)	5 (42)	0.67
Laboratory				
Creatinine (mg/dl; median [IQR])	1.11 (0.90, 1.61)	1.07 (1.00, 1.30)	1.45 (0.86, 1.89)	0.18
BNP (pg/ml; median [IQR])	545 (226, 1612)	297 (197, 843)	1385 (360, 1806)	0.17
Echocardiography				
Ejection fraction (%; mean ± SD)	47 ± 14	45 ± 13	49 ± 15	0.46
Aortic insufficiency* (mean ± SD)	1.1 ± 0.7	1.0 ± 0.7	1.1 ± 0.7	0.90
RVSP (mmHg; mean ± SD)	48 ± 17	43 ± 12	53 ± 19	0.17
RVSP >55 mmHg	7 (30)	2 (18)	5 (42)	0.37
Transfemoral approach	17 (74)	6 (55)	11 (92)	0.07

*All results are presented as n (%) unless otherwise noted.

BNP = brain natriuretic peptide, CABG = coronary artery bypass grafting, COPD = chronic obstructive pulmonary disease, IQR = interquartile range, NYHA = New York Heart Association, PCI = percutaneous coronary intervention, RVSP = right ventricular systolic pressure, STS-PROM = Society of Thoracic Surgeons predicted risk of mortality, TAVR = transcatheter aortic valve replacement.

**Table 2:** Patient characteristics at time of CMR*

	**All (n = 23)**	**RF < 20% (n = 11)**	**RF > 20% (n = 12)**	**P value**
Age (mean ± SD)	83 ± 6	83 ± 7	83 ± 5	0.82
NYHA class (mean ± SD)	3.5 ± 0.8	3.4 ± 0.9	3.7 ± 0.7	0.37
ΔNYHA class since TAVR (median[IQR])	1 (0, 1)	1 (−1, 1)	0 (0, 1)	0.77
STS-PROM (%; mean ± SD)	7.8 ± 4.6	6.3 ± 2.3	9.0 ± 5.7	0.16
ΔSTS-PROM since TAVR (%; median[IQR])	0 (−0.5, 1.7)	0 (−1.1, 0)	0.8 (−0.4, 3.0)	0.15
Laboratory				
Creatinine (mg/dl; median[IQR])	1.29 (1.00, 1.84)	1.11 (1.00, 1.37)	1.61 (1.06, 2.63)	0.011
ΔCreatinine since TAVR (mg/dl; median[IQR])	0.1 (−0.06, 0.39)	0.03 (−0.08, 0.19)	0.36 (0.10, 0.55)	0.13
BNP (pg/ml; median[IQR]))	496 (252, 1335)	432 (235, 546)	1038 (339, 1948)	0.05
ΔBNP since TAVR (pg/ml; median[IQR])	0 (−419, 199)	0 (−411, 85)	68 (−441, 240)	0.73
Echocardiography				
Circumferential extent (%; mean ± SD)	34.2 ± 18.4	23.3 ± 18.2	44.3 ± 11.9	0.003
CMR				
Ejection fraction (%; mean ± SD)	48 ± 12	47 ± 10	49 ± 14	0.78
Other valve disease greater than mild†	14 (61)	4 (36)	10 (83)	0.04

*All results are presented as n (%) unless otherwise noted.

†Includes mitral and tricuspid regurgitation.

BNP = brain natriuretic peptide, CMR = cardiovascular magnetic resonance, IQR = interquartile range, NYHA = New York Heart Association, PVL = paravalvular leak, RF = regurgitant fraction, STS-PROM = Society of Thoracic Surgeons predicted risk of mortality, TAVR = transcatheter aortic valve replacement, TTE = transthoracic echocardiography.

### Paravalvular leak classification by echocardiography and CMR

**Figure 3:**
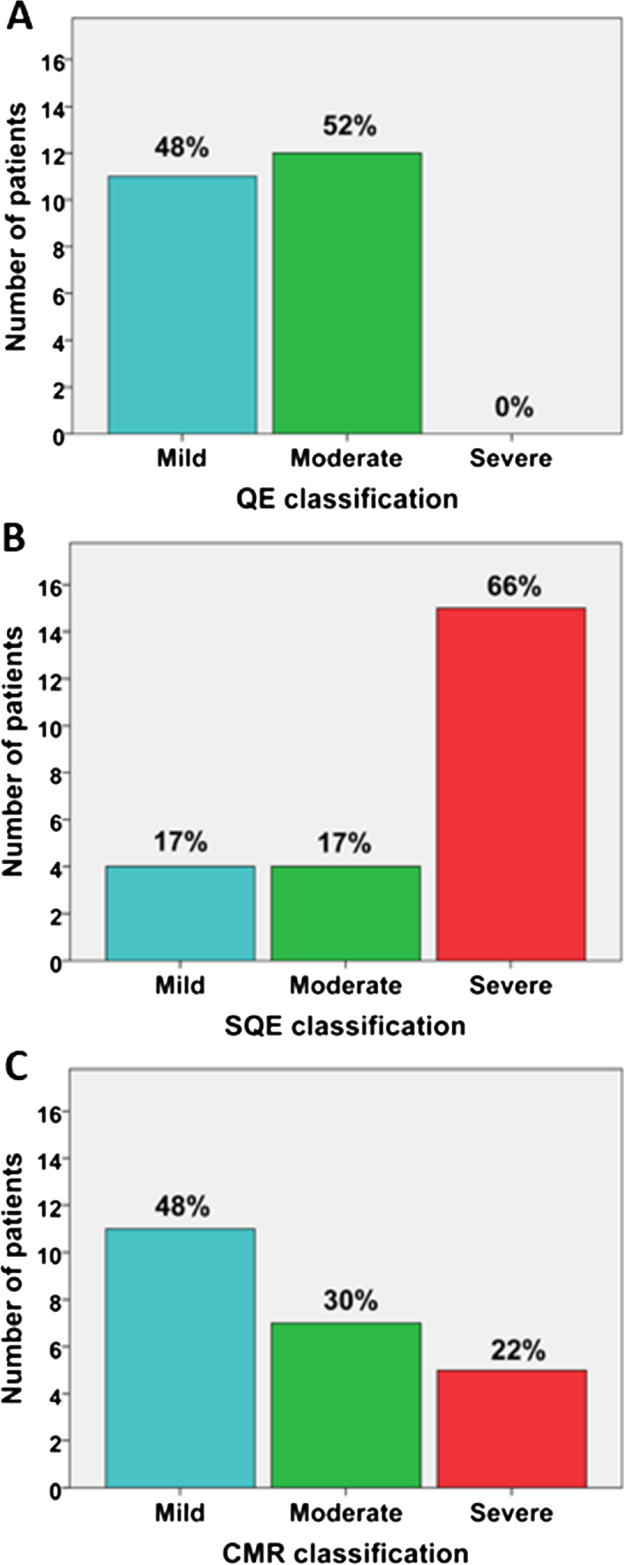
**Comparison of paravalvular leak (PVL) classification by qualitative echocardiography (QE), semi-quantitative echocardiography (SQE), and cardiovascular magnetic resonance (CMR).** QE classification included the estimated width of the color Doppler jet in the left ventricular outflow tract: mild (jet width < 25% of left ventricular outflow tract width), moderate (jet width 25 to 65% of left ventricular outflow tract width), and severe (jet width >65% of left ventricular outflow tract width). SQE included the circumferential extent as the sum of the paravalvular leak jet circumference(s) divided by the valve circumference: mild (<10%), moderate (10-30%), and severe (>30%). CMR classification included the aortic regurgitant fraction as calculated by dividing the reverse flow volume by the forward flow volume: mild (≤20%), moderate (21-39%), and severe (≥40%).

**Figure 4:**
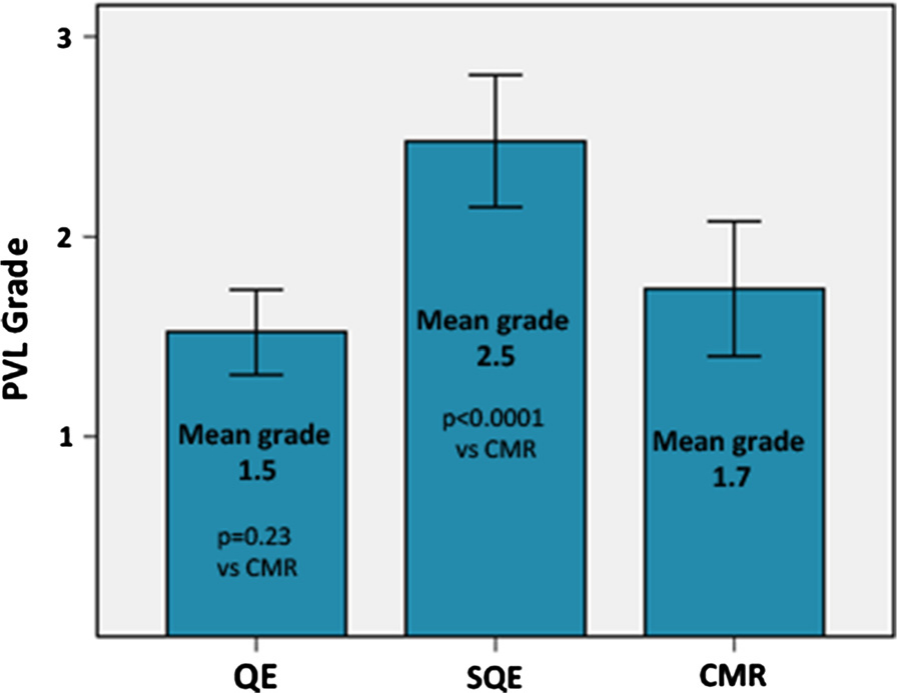
**Comparison of mean paravalvular leak (PVL) grade by qualitative echocardiography (QE), semi-quantitative echocardiography (SQE), and cardiovascular magnetic resonance (CMR).** PVL classification: mild = 1, moderate = 2, severe = 3. Error bars = 2 standard errors of the mean. P values are for paired-samples t-test.

All patients had adequate parasternal short-axis images on echocardiogram for semi-quantitative analysis. Suprasternal notch and subcostal views of the descending aorta were not consistently of diagnostic quality to assess diastolic flow reversal. Parasternal short and long-axis views of the right ventricular outflow tract and pulmonary artery were also not consistently of diagnostic quality to perform quantitative Doppler analysis. PVL classification by QE, SQE, and CMR is shown in Figure 3. Greater than mild PVL was present in 52%, 83% and 52% of patients by QE, SQE, and CMR, respectively. There was a poor correlation between QE and CMR (Spearman r = 0.26, p = 0.24) and a moderate correlation between SQE and CMR (Spearman r = 0.59; p = 0.003). Mean PVL severity was not significantly different by QE and CMR, however, SQE overestimated severity compared to CMR (see Figure 4). PVL was reclassified by CMR in a substantial number of patients compared with QE and SQE (see Table 3).

**Table 3:** **Number of patients in which the PVL grade was reclassified after performing CMR**

	**All n = 23 (%)**
Qualitative echocardiography reclassification	11 (48)
Upgrade	7 (31)
Downgrade	4 (17)
Semi-quantitative echocardiography reclassification	13 (57)
Upgrade	0 (0)
Downgrade	13 (57)

CMR = cardiovascular magnetic resonance, PVL paravalvular leak, RF = regurgitant fraction, TTE = transthoracic echocardiography.

### Primary composite outcome

**Figure 5:**
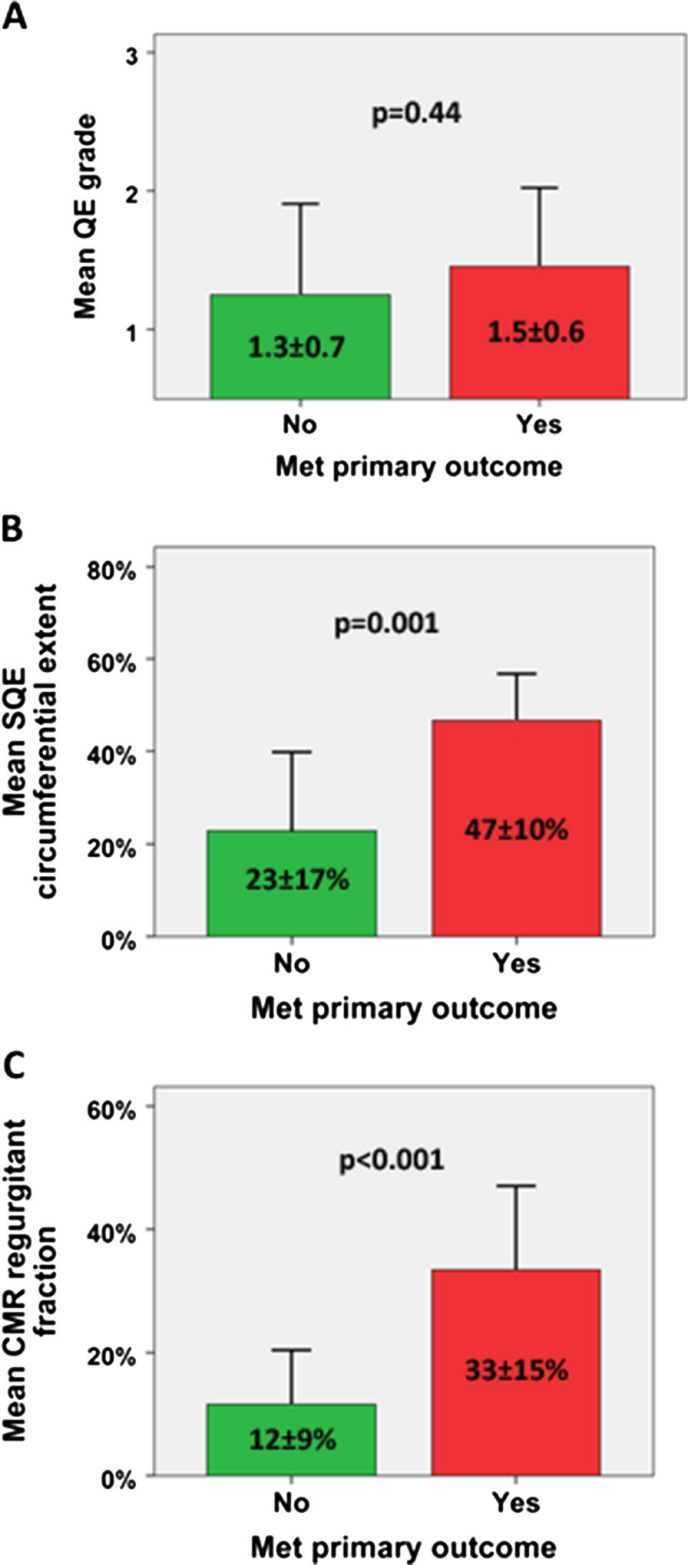
**Comparison of paravalvular leak (PVL) severity in patients with different primary composite outcomes by imaging method. (A)** Qualitative echocardiography (QE): 1 = mild, 2 = moderate, 3 = severe. **(B)** Semi-quantitative echocardiography (SQE) circumferential extent: mild (<10%), moderate (10-30%), and severe (>30%). **(C)** Cardiovascular magnetic resonance (CMR) regurgitant fraction: mild (≤20%), moderate (21-39%), and severe (≥40%). Primary composite outcome = repeat invasive therapy, heart failure hospitalization, and all-cause death. P values are for independent samples t-test.

**Figure 6:**
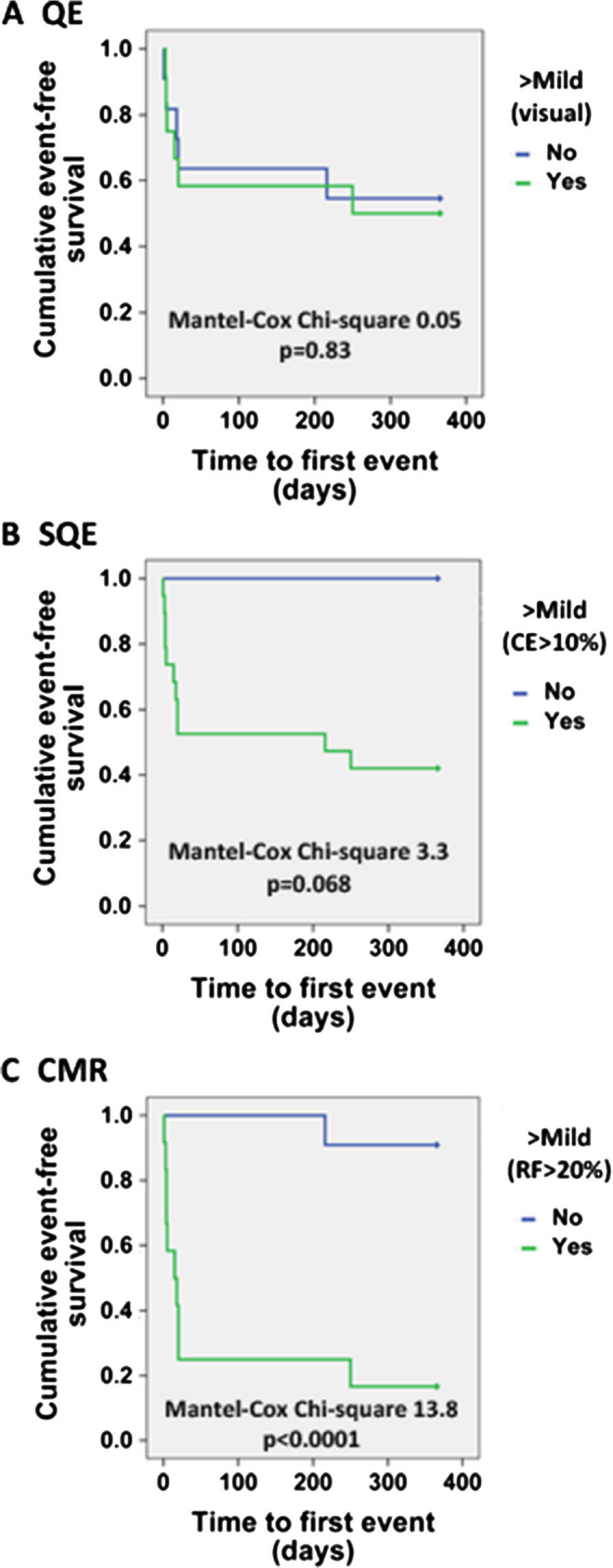
**Primary composite outcome Kaplan-Meier survival analysis for patients with greater than mild paravalvular leak (PVL) by imaging method.** QE = qualitative echocardiography, SQE = semi-quantitative echocardiography, CMR = cardiovascular magnetic resonance. CE = circumferential extent. Primary composite outcome = repeat invasive therapy, heart failure hospitalization, and all-cause death.

At one year (mean 11.5 ± 1.4 months), 48% of the patients met the primary composite outcome of all-cause death, heart failure hospitalization, and intractable heart failure symptoms necessitating repeat invasive therapy (see Table 4). The average time to first event was 51 days (9 days for repeat invasive therapy, 97 days for heart failure admission, and 118 days for all-cause death). Patients who experienced the primary outcome had significantly higher serum creatinine levels at the time of CMR (1.9 ± 0.80 mg/dl versus 1.17 ± 0.32 mg/dl, p = 0.008) and higher EF by CMR (53 ± 10% versus 44 ± 12%, p = 0.048). The primary outcome occurred in 9%, 71%, and 100% of patients with mild, moderate, and severe PVL by CMR, respectively. Patients with greater than mild PVL by CMR were more likely to experience the primary outcome compared to those with mild or less PVL (p = 0.001), while patients with greater than mild PVL by QE or SQE were not (p = 1.0 and p = 0.093, respectively). Otherwise, there were no significant baseline differences in patients with and without a primary outcome event. Severity of PVL by QE, SQE, and CMR in those with and without a primary outcome event is shown in Figure 5. Kaplan-Meier survival analysis stratified by greater than mild PVL stratified by QE, SQE, and CMR is shown in Figure 6.

**Table 4:** **Follow-up Primary Composite Outcomes**

	**All n = 23 (%)**	**RF ≤ 20% n = 11 (%)**	**RF > 20% n = 12 (%)**	P value*
Outcomes	11 (48)	1 (9)	10 (83)	0.001
Intractable heart failure symptoms necessitating repeat invasive therapy	8 (35)	0 (0)	8 (67)	
Heart failure hospitalization	4 (17)	0 (0)	4 (33)	
All-cause death	5 (22)	1 (9)	4 (33)	

*For Fisher’s exact test.

RF = cardiovascular magnetic resonance derived regurgitant fraction.

### Secondary composite outcome

**Figure 7:**
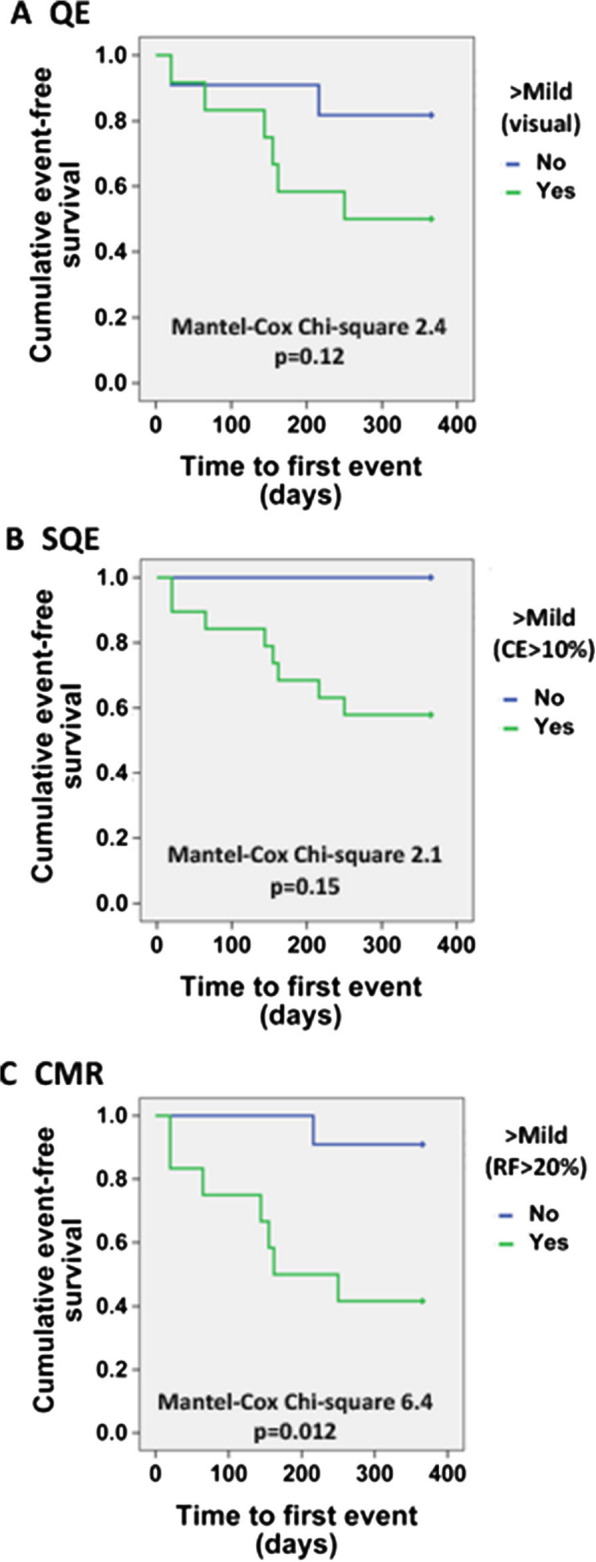
**Secondary composite outcome Kaplan-Meier survival analysis for patients with greater than mild paravalvular leak (PVL) by imaging method.** QE = qualitative echocardiography, SQE = semi-quantitative echocardiography, CMR = cardiovascular magnetic resonance. CE = circumferential extent. Secondary composite outcome = heart failure hospitalization and all-cause death.

At one year, 35% of patients met the secondary composite outcome of all-cause death and heart failure hospitalization. The average time to heart failure admission was 109 days and average time to death was 150 days. The secondary outcome occurred in 9%, 57%, and 60% of patients with mild, moderate, and severe PVL by CMR, respectively. Patients with greater than mild PVL by CMR were significantly more likely to experience a secondary composite outcome event compared to those with mild or less PVL (p = 0.027), while patients with greater than mild PVL by QE or SQE were not (p = 0.19 and p = 0.26, respectively). Otherwise, there were no significant differences in patients with and without a secondary outcome event. Kaplan-Meier survival analysis stratified by greater than mild PVL by QE, SQE, and CMR is shown in Figure 7.

### Reproducibility analysis

Inter-observer variability for QE, SQE and CMR was assessed by 2 independent readers (G.H. and S.L.) in 10 randomly selected patients for each method. For QE, the intraclass correlation coefficient for PVL severity by visual grading was 0.61 (p = 0.022), with excellent agreement between readers for PVL classification (Cohen’s kappa coefficient 1.0, p = 0.002). For SQE, the intraclass correlation coefficient for PVL circumferential extent by the VARC II method was 0.58 (p = 0.030), with good agreement between readers for PVL classification (Cohen’s kappa coefficient 0.60, p = 0.016). For CMR, the intraclass correlation coefficient for PVL regurgitant fraction was 0.93 (p < 0.0001) with excellent agreement between readers for PVL classification (Cohen’s kappa coefficient 0.82, p < 0.0001).

## Discussion

We present an early experience demonstrating the added value of CMR for diagnostic classification and risk stratification in symptomatic patients with post-TAVR PVL. PVL classification by PCMR flow quantification was reproducible and provided prognostic value superior to both qualitative and semi-quantitative echocardiography, suggesting that more widespread use of CMR derived PVL quantification may be warranted in symptomatic post-TAVR patients. Flow quantification by PCMR is a well-established method for evaluating proximal aortic forward and reverse volumes [7,8]. Aortic regurgitation quantification is more reproducible by CMR compared to echocardiography for native valve disease [13,14], however, it is noteworthy that quantitative RF values of aortic regurgitation severity obtained by PCMR are systematically lower than those obtained by echocardiography [15,16]. CMR derived quantitative findings have also shown prognostic value in native aortic regurgitation patients [17]. We present the first study evaluating the prognostic value of CMR derived PVL quantification in post-TAVR patients, in addition to diagnostic reclassification of PVL by CMR relative to various echocardiographic techniques.

CMR has demonstrated feasibility for the quantification of post-TAVR PVL [9-11]. In contrast to native aortic valve regurgitation, post-TAVR PVL is underestimated by QE compared to CMR [10,18,19]. Sherif et al. initially showed that QE underestimated the degree of PVL by ≥1 grade in 7 of 16 (44%) patients compared to CMR [10]. Recently, a multiparametric TTE approach was found to underestimate the degree of PVL by ≥1 grade relative to CMR to a varying extent: 15 of 65 (23%) patients in Orwat et al. [18] and 26 of 42 (62%) patients in Ribiero et al. [19]. These findings are consistent with our study, in which QE identified no cases of severe PVL and PVL severity was upgraded by CMR by ≥1 grade in nearly one-third of patients. A notable difference between our study and those above is that they utilized routine CMR screening in post-TAVR patients regardless of symptoms, while our study evaluated CMR as a strategy to clarify post-TAVR symptoms. Importantly, we demonstrated limited prognostic power of QE while CMR was a strong predictor of adverse events, which highlights the clinical importance of the discrepancies between PVL grading with these modalities.

It is generally accepted that greater than mild PVL by echocardiography is associated with worse patient outcomes post-TAVR [3,4]. However, both transthoracic and transesophageal echocardiography may have significant limitations for the characterization and quantification of post-TAVR PVL. These limitations are related to two-dimensional anatomic views, angle dependence of Doppler assessment, multiple eccentric jets, and signal attenuation due to native calcification and implanted prosthetic material. Any combination of these factors can result in PVL underestimation. Such underestimation of post-TAVR PVL by QE may have led to previous findings implying that even mild post-TAVR PVL is associated with worse outcomes [1]. In our study, PVL severity by QE did not predict patient outcomes, further highlighting the limitations of QE in risk stratifying post-TAVR PVL. The assessment of PVL by PCMR overcomes the limitations of anatomic views and visual assessment to provide a truly quantitative evaluation of retrograde diastolic flow in the proximal aorta. Importantly, PCMR is technically feasible for PVL assessment in the balloon-expandable and self-expanding valves currently approved for use in the United States [10].

In an attempt to improve echocardiographic analysis, SQE has been performed in addition to QE. However, there is concern that PVL classification by SQE circumferential extent per VARC II criteria overestimates post-TAVR PVL severity as compared to quantitative Doppler [6,19,20] and CMR quantification [21]. Although technical limitations precluded routine quantitative Doppler in our series of symptomatic patients, we also found overestimation of PVL severity by SQE compared to CMR, with greater than 80% of patients classified as having greater than mild PVL by SQE compared to 48% by CMR. These findings are in concordance with those of Ribiero et al. [19], who found that 38% of their asymptomatic post-TAVR patients had greater than mild PVL by SQE compared to 24% by CMR. Our study also demonstrated inferior prognostic power of SQE compared to CMR.

Our study demonstrates that greater than mild post-TAVR PVL by CMR is associated with a worse prognosis in a symptomatic population. This finding is supported by data from Merten *et al.* demonstrating that patients with no or mild post-TAVR PVL by CMR undergo beneficial LV structural remodeling, while those with greater than mild PVL do not [9]. A left ventricle that has chronically remodeled to facilitate pressure overload from aortic stenosis is expected to poorly tolerate moderate or severe regurgitation. Symptoms related to post-TAVR PVL might be difficult to differentiate from those related to systolic or diastolic heart failure and we believe CMR adds incremental value in this situation [22]. Our initial findings utilizing highly reproducible CMR quantification support further prospective validation of CMR for the evaluation and prognostication of post-TAVR PVL in a larger population of TAVR patients.

### Limitations

The primary limitations of our study are the small number of patients from a single institution and retrospective collection of data. Small sample size may contribute to limited power to draw conclusive results, however this issue may be mitigated by the high event rate (48%) for the primary composite outcome in this highly symptomatic cohort. The inclusion of symptomatic post-TAVR patients from our single institution also limits external validity, so generalization beyond our sample must be done with caution. Retrospective analysis may not account for unidentified confounders associated with poor outcomes post-TAVR, however, all patients were taking part in parallel prospective TAVR trials or registries in which meticulous follow-up and data collection were ensured.

Our primary composite outcome included repeat invasive therapy for refractory heart failure symptoms due to PVL, which could be driven by CMR findings and thus cause post-test bias. The secondary composite outcome, which did not include repeat invasive therapy, was significant for patients with >20% RF by CMR. Many of our patients had difficult or suboptimal echocardiography. Semi-quantitative assessment using descending aortic flow reversal and quantitative assessment using right ventricular outflow tract or pulmonary artery Doppler were not routinely performed. This deficit likely reflects a modality specific shortcoming, as even experienced echocardiography centers report a substantial rate of technically difficult routine studies [23].

## Conclusions

CMR stratifies post-TAVR PVL severity and should be considered early in the evaluation of symptomatic post-TAVR patients. CMR is highly reproducible and commonly reclassifies PVL grade compared with qualitative and semi-quantitative TTE. Patients with greater than mild PVL by CMR (RF > 20%) had a higher incidence of the primary composite outcome of all-cause death, heart failure hospitalization and intractable heart failure symptoms necessitating repeat invasive therapy.
